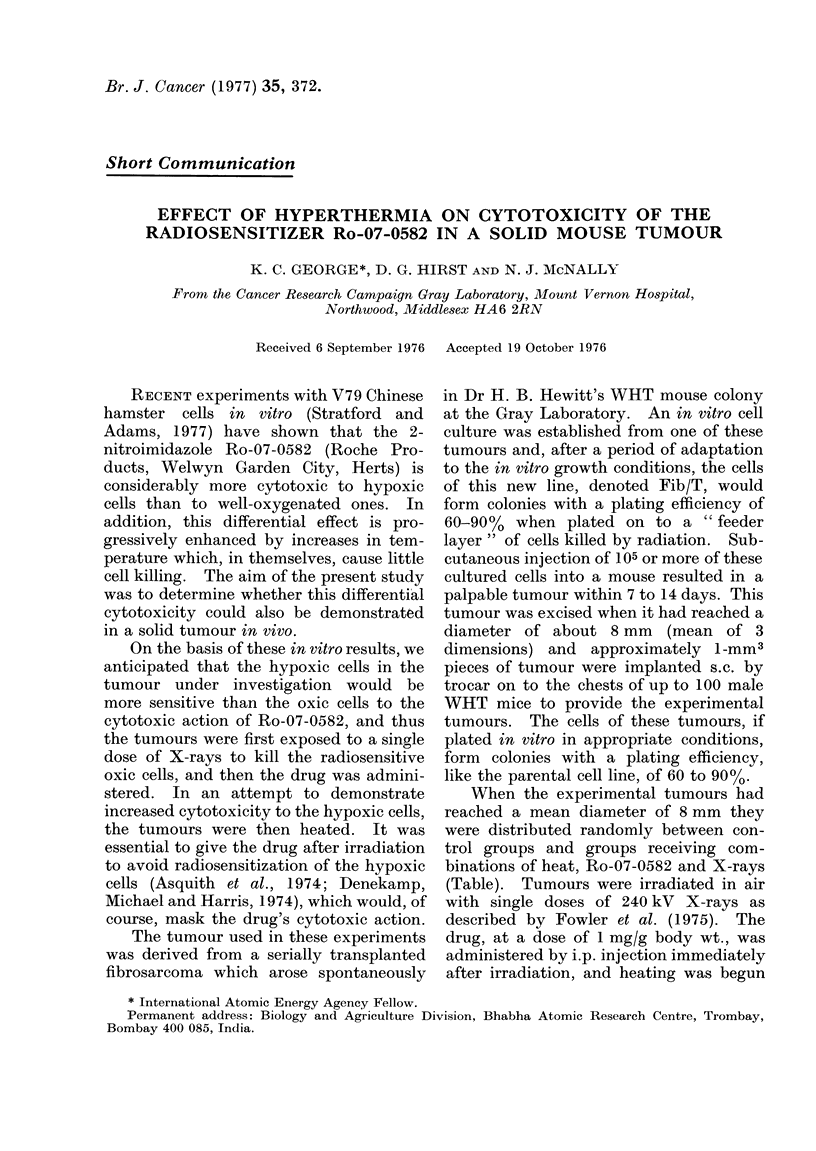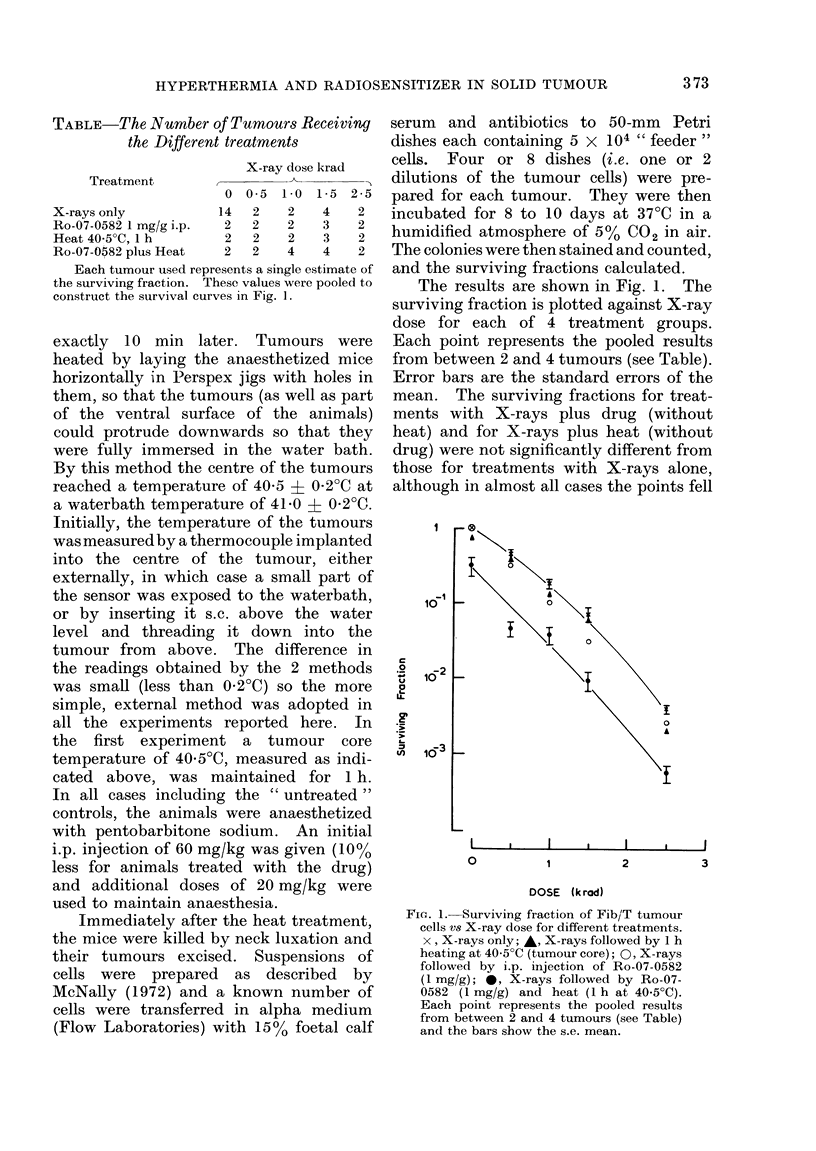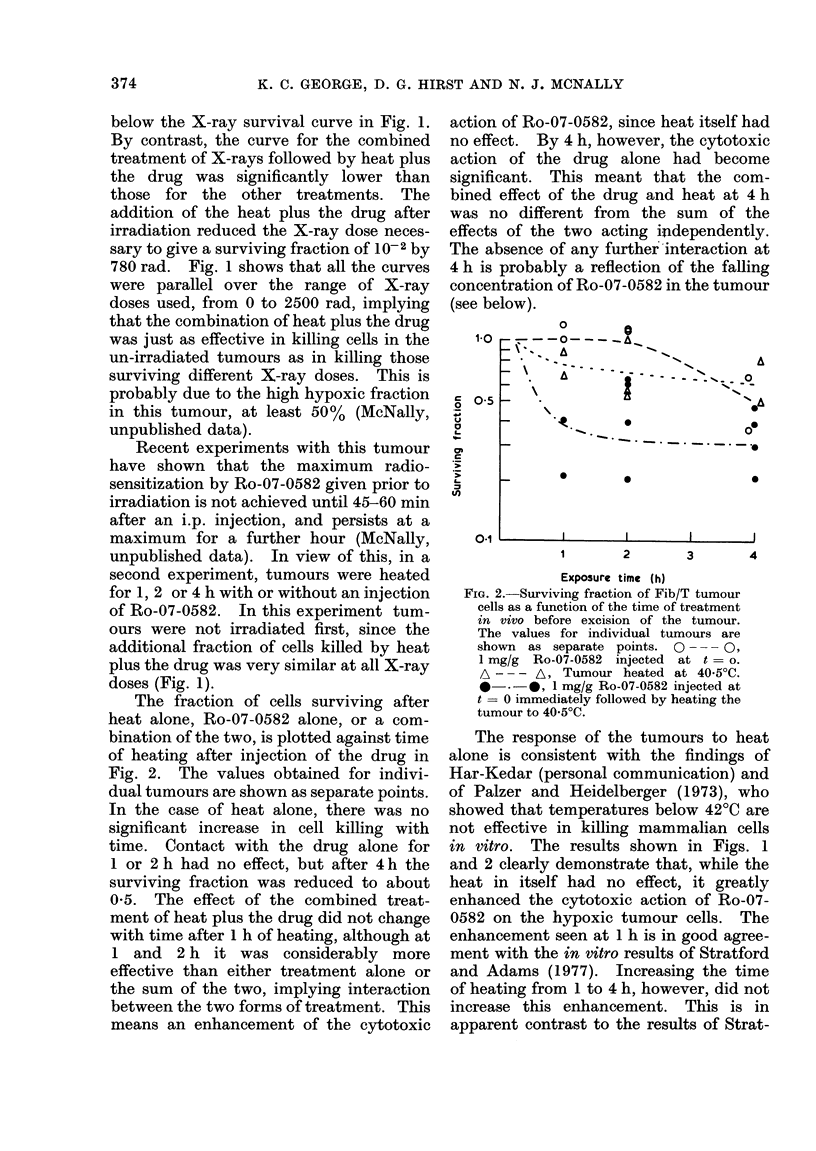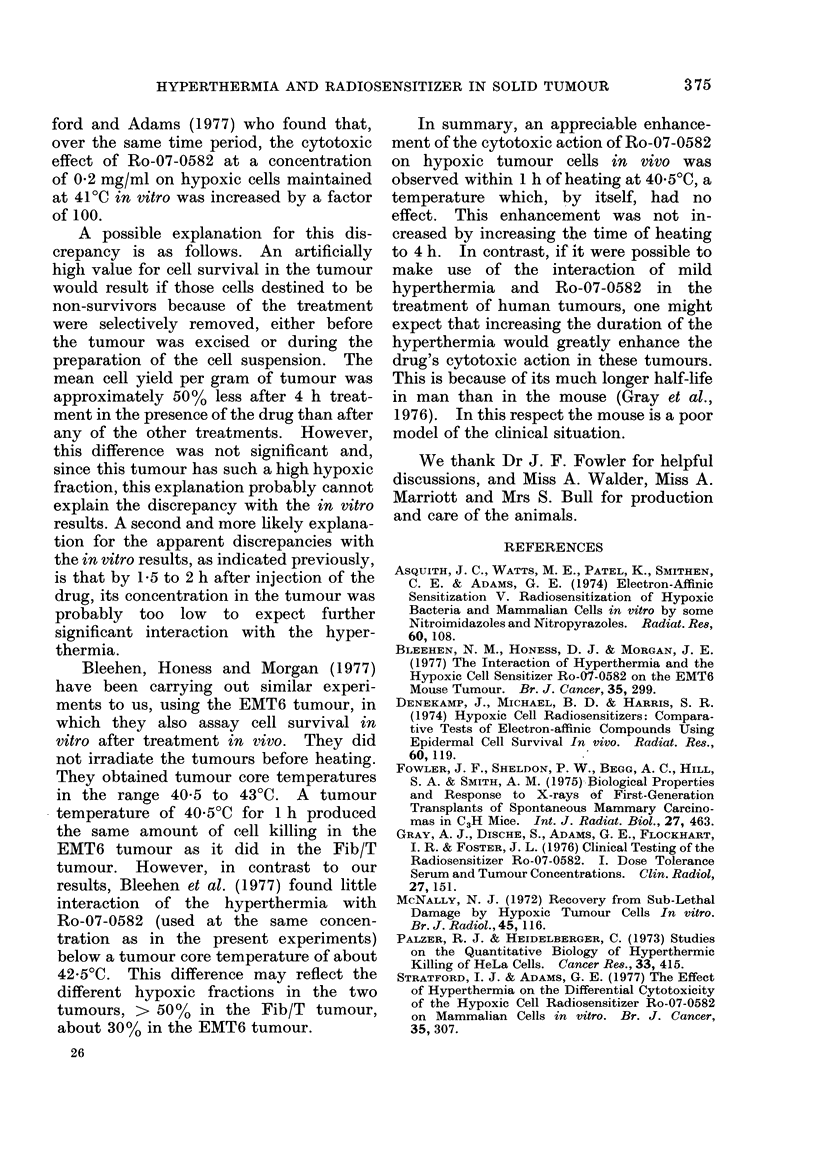# Effect of hyperthermia on cytotoxicity of the radiosensitizer Ro-07-0582 in a solid mouse tumour.

**DOI:** 10.1038/bjc.1977.52

**Published:** 1977-03

**Authors:** K. C. George, D. G. Hirst, N. J. McNally


Br. J. Cancer (1977) 35, 372.

### Short Communication

### EFFECT OF HYPERTHERMIA ON CYTOTOXICITY OF THE RADIOSENSITIZER Ro-07-0582 IN A SOLID MOUSE TUMOUR

K. C. GEORGE*, D. G. HIRST AND N. J. McNALLY

*From the Cancer Research Campaign Gray Laboratory, Mount Vernon Hospital, Northwood, Middlesex HA6 2RN*

Received 6 September 1976 Accepted 19 October 1976

RECENT experiments with V79 Chinese hamster cells *in vitro* (Stratford and Adams, 1977) have shown that the 2-nitroimidazole Ro-07-0582 (Roche Products, Welwyn Garden City, Herts) is considerably more cytotoxic to hypoxic cells than to well-oxygenated ones. In addition, this differential effect is progressively enhanced by increases in temperature which, in themselves, cause little cell killing. The aim of the present study was to determine whether this differential cytotoxicity could also be demonstrated in a solid tumour *in vivo*.

On the basis of these *in vitro* results, we anticipated that the hypoxic cells in the tumour under investigation would be more sensitive than the oxic cells to the cytotoxic action of Ro-07-0582, and thus the tumours were first exposed to a single dose of X-rays to kill the radiosensitive oxic cells, and then the drug was administered. In an attempt to demonstrate increased cytotoxicity to the hypoxic cells, the tumours were then heated. It was essential to give the drug after irradiation to avoid radiosensitization of the hypoxic cells (Asquith *et al.*, 1974; Denekamp, Michael and Harris, 1974), which would, of course, mask the drug's cytotoxic action.

The tumour used in these experiments was derived from a serially transplanted fibrosarcoma which arose spontaneously in Dr H. B. Hewitt's WHT mouse colony at the Gray Laboratory. An *in vitro* cell culture was established from one of these tumours and, after a period of adaptation to the *in vitro* growth conditions, the cells of this new line, denoted Fib/T, would form colonies with a plating efficiency of 60–90% when plated on to a "feeder layer" of cells killed by radiation. Subcutaneous injection of $10^5$ or more of these cultured cells into a mouse resulted in a palpable tumour within 7 to 14 days. This tumour was excised when it had reached a diameter of about 8 mm (mean of 3 dimensions) and approximately 1-mm$^3$ pieces of tumour were implanted s.c. by trocar on to the chests of up to 100 male WHT mice to provide the experimental tumours. The cells of these tumours, if plated *in vitro* in appropriate conditions, form colonies with a plating efficiency, like the parental cell line, of 60 to 90%.

When the experimental tumours had reached a mean diameter of 8 mm they were distributed randomly between control groups and groups receiving combinations of heat, Ro-07-0582 and X-rays (Table). Tumours were irradiated in air with single doses of 240 kV X-rays as described by Fowler *et al.* (1975). The drug, at a dose of 1 mg/g body wt., was administered by i.p. injection immediately after irradiation, and heating was begun

* International Atomic Energy Agency Fellow.
Permanent address: Biology and Agriculture Division, Bhabha Atomic Research Centre, Trombay, Bombay 400 085, India.

TABLE—*The Number of Tumours Receiving the Different treatments*

| Treatment | X-ray dose krad | | | | |
|---|---|---|---|---|---|
| | 0 | 0·5 | 1·0 | 1·5 | 2·5 |
| X-rays only | 14 | 2 | 2 | 4 | 2 |
| Ro-07-0582 1 mg/g i.p. | 2 | 2 | 2 | 3 | 2 |
| Heat 40·5°C, 1 h | 2 | 2 | 2 | 3 | 2 |
| Ro-07-0582 plus Heat | 2 | 2 | 4 | 4 | 2 |

Each tumour used represents a single estimate of the surviving fraction. These values were pooled to construct the survival curves in Fig. 1.

exactly 10 min later. Tumours were heated by laying the anaesthetized mice horizontally in Perspex jigs with holes in them, so that the tumours (as well as part of the ventral surface of the animals) could protrude downwards so that they were fully immersed in the water bath. By this method the centre of the tumours reached a temperature of 40·5 ± 0·2°C at a waterbath temperature of 41·0 ± 0·2°C. Initially, the temperature of the tumours was measured by a thermocouple implanted into the centre of the tumour, either externally, in which case a small part of the sensor was exposed to the waterbath, or by inserting it s.c. above the water level and threading it down into the tumour from above. The difference in the readings obtained by the 2 methods was small (less than 0·2°C) so the more simple, external method was adopted in all the experiments reported here. In the first experiment a tumour core temperature of 40·5°C, measured as indicated above, was maintained for 1 h. In all cases including the " untreated " controls, the animals were anaesthetized with pentobarbitone sodium. An initial i.p. injection of 60 mg/kg was given (10% less for animals treated with the drug) and additional doses of 20 mg/kg were used to maintain anaesthesia.

Immediately after the heat treatment, the mice were killed by neck luxation and their tumours excised. Suspensions of cells were prepared as described by McNally (1972) and a known number of cells were transferred in alpha medium (Flow Laboratories) with 15% foetal calf serum and antibiotics to 50-mm Petri dishes each containing 5 × $10^4$ " feeder " cells. Four or 8 dishes (*i.e.* one or 2 dilutions of the tumour cells) were prepared for each tumour. They were then incubated for 8 to 10 days at 37°C in a humidified atmosphere of 5% $CO_2$ in air. The colonies were then stained and counted, and the surviving fractions calculated.

The results are shown in Fig. 1. The surviving fraction is plotted against X-ray dose for each of 4 treatment groups. Each point represents the pooled results from between 2 and 4 tumours (see Table). Error bars are the standard errors of the mean. The surviving fractions for treatments with X-rays plus drug (without heat) and for X-rays plus heat (without drug) were not significantly different from those for treatments with X-rays alone, although in almost all cases the points fell

FIG. 1.—Surviving fraction of Fib/T tumour cells *vs* X-ray dose for different treatments. ×, X-rays only; ▲, X-rays followed by 1 h heating at 40·5°C (tumour core); ○, X-rays followed by i.p. injection of Ro-07-0582 (1 mg/g); ●, X-rays followed by Ro-07-0582 (1 mg/g) and heat (1 h at 40·5°C). Each point represents the pooled results from between 2 and 4 tumours (see Table) and the bars show the s.e. mean.

below the X-ray survival curve in Fig. 1. By contrast, the curve for the combined treatment of X-rays followed by heat plus the drug was significantly lower than those for the other treatments. The addition of the heat plus the drug after irradiation reduced the X-ray dose necessary to give a surviving fraction of $10^{-2}$ by 780 rad. Fig. 1 shows that all the curves were parallel over the range of X-ray doses used, from 0 to 2500 rad, implying that the combination of heat plus the drug was just as effective in killing cells in the un-irradiated tumours as in killing those surviving different X-ray doses. This is probably due to the high hypoxic fraction in this tumour, at least 50% (McNally, unpublished data).

Recent experiments with this tumour have shown that the maximum radiosensitization by Ro-07-0582 given prior to irradiation is not achieved until 45–60 min after an i.p. injection, and persists at a maximum for a further hour (McNally, unpublished data). In view of this, in a second experiment, tumours were heated for 1, 2 or 4 h with or without an injection of Ro-07-0582. In this experiment tumours were not irradiated first, since the additional fraction of cells killed by heat plus the drug was very similar at all X-ray doses (Fig. 1).

The fraction of cells surviving after heat alone, Ro-07-0582 alone, or a combination of the two, is plotted against time of heating after injection of the drug in Fig. 2. The values obtained for individual tumours are shown as separate points. In the case of heat alone, there was no significant increase in cell killing with time. Contact with the drug alone for 1 or 2 h had no effect, but after 4 h the surviving fraction was reduced to about 0·5. The effect of the combined treatment of heat plus the drug did not change with time after 1 h of heating, although at 1 and 2 h it was considerably more effective than either treatment alone or the sum of the two, implying interaction between the two forms of treatment. This means an enhancement of the cytotoxic action of Ro-07-0582, since heat itself had no effect. By 4 h, however, the cytotoxic action of the drug alone had become significant. This meant that the combined effect of the drug and heat at 4 h was no different from the sum of the effects of the two acting independently. The absence of any further interaction at 4 h is probably a reflection of the falling concentration of Ro-07-0582 in the tumour (see below).

FIG. 2.—Surviving fraction of Fib/T tumour cells as a function of the time of treatment *in vivo* before excision of the tumour. The values for individual tumours are shown as separate points. ○ – – – ○, 1 mg/g Ro-07-0582 injected at $t$ = o. △ – – – △, Tumour heated at 40·5°C. ●—·—●, 1 mg/g Ro-07-0582 injected at $t$ = 0 immediately followed by heating the tumour to 40·5°C.

The response of the tumours to heat alone is consistent with the findings of Har-Kedar (personal communication) and of Palzer and Heidelberger (1973), who showed that temperatures below 42°C are not effective in killing mammalian cells *in vitro*. The results shown in Figs. 1 and 2 clearly demonstrate that, while the heat in itself had no effect, it greatly enhanced the cytotoxic action of Ro-07-0582 on the hypoxic tumour cells. The enhancement seen at 1 h is in good agreement with the *in vitro* results of Stratford and Adams (1977). Increasing the time of heating from 1 to 4 h, however, did not increase this enhancement. This is in apparent contrast to the results of Stratford and Adams (1977) who found that, over the same time period, the cytotoxic effect of Ro-07-0582 at a concentration of 0·2 mg/ml on hypoxic cells maintained at 41°C *in vitro* was increased by a factor of 100.

A possible explanation for this discrepancy is as follows. An artificially high value for cell survival in the tumour would result if those cells destined to be non-survivors because of the treatment were selectively removed, either before the tumour was excised or during the preparation of the cell suspension. The mean cell yield per gram of tumour was approximately 50% less after 4 h treatment in the presence of the drug than after any of the other treatments. However, this difference was not significant and, since this tumour has such a high hypoxic fraction, this explanation probably cannot explain the discrepancy with the *in vitro* results. A second and more likely explanation for the apparent discrepancies with the *in vitro* results, as indicated previously, is that by 1·5 to 2 h after injection of the drug, its concentration in the tumour was probably too low to expect further significant interaction with the hyperthermia.

Bleehen, Honess and Morgan (1977) have been carrying out similar experiments to us, using the EMT6 tumour, in which they also assay cell survival *in vitro* after treatment *in vivo*. They did not irradiate the tumours before heating. They obtained tumour core temperatures in the range 40·5 to 43°C. A tumour temperature of 40·5°C for 1 h produced the same amount of cell killing in the EMT6 tumour as it did in the Fib/T tumour. However, in contrast to our results, Bleehen *et al.* (1977) found little interaction of the hyperthermia with Ro-07-0582 (used at the same concentration as in the present experiments) below a tumour core temperature of about 42·5°C. This difference may reflect the different hypoxic fractions in the two tumours, > 50% in the Fib/T tumour, about 30% in the EMT6 tumour.

In summary, an appreciable enhancement of the cytotoxic action of Ro-07-0582 on hypoxic tumour cells *in vivo* was observed within 1 h of heating at 40·5°C, a temperature which, by itself, had no effect. This enhancement was not increased by increasing the time of heating to 4 h. In contrast, if it were possible to make use of the interaction of mild hyperthermia and Ro-07-0582 in the treatment of human tumours, one might expect that increasing the duration of the hyperthermia would greatly enhance the drug's cytotoxic action in these tumours. This is because of its much longer half-life in man than in the mouse (Gray *et al.*, 1976). In this respect the mouse is a poor model of the clinical situation.

We thank Dr J. F. Fowler for helpful discussions, and Miss A. Walder, Miss A. Marriott and Mrs S. Bull for production and care of the animals.

### REFERENCES

ASQUITH, J. C., WATTS, M. E., PATEL, K., SMITHEN, C. E. & ADAMS, G. E. (1974) Electron-Affinic Sensitization V. Radiosensitization of Hypoxic Bacteria and Mammalian Cells *in vitro* by some Nitroimidazoles and Nitropyrazoles. *Radiat. Res*, **60**, 108.

BLEEHEN, N. M., HONESS, D. J. & MORGAN, J. E. (1977) The Interaction of Hyperthermia and the Hypoxic Cell Sensitizer Ro-07-0582 on the EMT6 Mouse Tumour. *Br. J. Cancer*, **35**, 299.

DENEKAMP, J., MICHAEL, B. D. & HARRIS, S. R. (1974) Hypoxic Cell Radiosensitizers: Comparative Tests of Electron-affinic Compounds Using Epidermal Cell Survival *In vivo*. *Radiat. Res.*, **60**, 119.

FOWLER, J. F., SHELDON, P. W., BEGG, A. C., HILL, S. A. & SMITH, A. M. (1975) Biological Properties and Response to X-rays of First-Generation Transplants of Spontaneous Mammary Carcinomas in $C_3H$ Mice. *Int. J. Radiat. Biol.*, **27**, 463.

GRAY, A. J., DISCHE, S., ADAMS, G. E., FLOCKHART, I. R. & FOSTER, J. L. (1976) Clinical Testing of the Radiosensitizer Ro-07-0582. I. Dose Tolerance Serum and Tumour Concentrations. *Clin. Radiol*, **27**, 151.

MCNALLY, N. J. (1972) Recovery from Sub-Lethal Damage by Hypoxic Tumour Cells *In vitro*. *Br. J. Radiol.*, **45**, 116.

PALZER, R. J. & HEIDELBERGER, C. (1973) Studies on the Quantitative Biology of Hyperthermic Killing of HeLa Cells. *Cancer Res.*, **33**, 415.

STRATFORD, I. J. & ADAMS, G. E. (1977) The Effect of Hyperthermia on the Differential Cytotoxicity of the Hypoxic Cell Radiosensitizer Ro-07-0582 on Mammalian Cells *in vitro*. *Br. J. Cancer*, **35**, 307.